# “CHildren with Inherited Platelet disorders Surveillance” (CHIPS) retrospective and prospective observational cohort study by Italian Association of Pediatric Hematology and Oncology (AIEOP)

**DOI:** 10.3389/fped.2022.967417

**Published:** 2022-11-22

**Authors:** Giuseppe Lassandro, Valentina Palladino, Michela Faleschini, Angelica Barone, Gianluca Boscarol, Simone Cesaro, Elena Chiocca, Piero Farruggia, Fiorina Giona, Chiara Gorio, Angela Maggio, Maddalena Marinoni, Antonio Marzollo, Giuseppe Palumbo, Giovanna Russo, Paola Saracco, Marco Spinelli, Federico Verzegnassi, Francesca Morga, Anna Savoia, Paola Giordano

**Affiliations:** ^1^ Interdisciplinary Department of Medicine, Pediatric Section, University of Bari “Aldo Moro”, Bari, Italy; ^2^ Department of Medical Genetics, Institute for Maternal and Child Health—IRCCS Burlo Garofolo, Trieste, Italy; ^3^ Pediatric Hematology Oncology, Dipartimento Materno-Infantile, Azienda Ospedaliero-Universitaria di Parma, Parma, Italy; ^4^ Department of Pediatrics, Central Teaching Hospital of Bolzano/Bozen, Bolzano, Italy; ^5^ Pediatric Hematology Oncology, Department of Mother and Child, Azienda Ospedaliera Universitaria Integrata Verona, Verona, Italy; ^6^ Pediatric Hematology Oncology, Department of Pediatric Hematology/Oncology and HSCT, Meyer Children's University Hospital, Florence, Italy; ^7^ Pediatric Hematology and Oncology Unit, ARNAS (Azienda di Rilievo Nazionale ad Alta Specializzazione) Ospedale Civico, Palermo, Italy; ^8^ Department of Translational and Precision Medicine, Sapienza University of Rome, AOU Policlinico Umberto I, Rome, Italy; ^9^ Hematology Oncology Unit, Children’s Hospital, ASST Spedali Civili, Brescia, Italy; ^10^ UOC Oncoematologia Pediatrica—IRCCS Ospedale Casa Sollievo Della Sofferenza, San Giovanni Rotondo, Italy; ^11^ Pediatric Hematology Oncology, Department of Mother and Child, Azienda Socio Sanitaria Settelaghi, Varese, Italy; ^12^ Pediatric Hematology, Oncology and Stem Cell Transplant Division, Padua University Hospital, Padua, Italy; ^13^ Department of Pediatric Hematology and Oncology Cell and Gene Therapy, Bambino Gesù Children's Hospital, IRCCS, Rome, Italy; ^14^ Department of Systems Medicine, University of Rome Tor Vergata, Rome, Italy; ^15^ Pediatric Hematology Oncology, Department of Clinical and Experimental Medicine, University of Catania, Catania, Italy; ^16^ Pediatric Hematology, Department of Pediatrics, University Hospital Città Della Salute e Della Scienza, Turin, Italy; ^17^ Pediatric Hematology Oncology, Department of Pediatrics, MBBM Foundation, Monza, Italy; ^18^ Department of Medical Sciences, University of Trieste, Trieste, Italy

**Keywords:** inherited thrombocytopenia, platelet, bleeding diseases/disorders, children, congenital thrombocytopenia

## Abstract

**Abstract:**

**Background:**

Inherited thrombocytopenias (ITs) are rare congenital bleeding disorders characterized by different clinical expression and variable prognosis. ITs are poorly known by clinicians and often misdiagnosed with most common forms of thrombocytopenia.

**Material and methods:**

“CHildren with Inherited Platelet disorders Surveillance” study (CHIPS) is a retrospective – prospective observational cohort study conducted between January 2003 and January 2022 in 17 centers affiliated to the Italian Association of Pediatric Hematology and Oncology (AIEOP). The primary objective of this study was to collect clinical and laboratory data on Italian pediatric patients with inherited thrombocytopenias. Secondary objectives were to calculate prevalence of ITs in Italian pediatric population and to assess frequency and genotype–phenotype correlation of different types of mutations in our study cohort.

**Results:**

A total of 139 children, with ITs (82 male - 57 female) were enrolled. ITs prevalence in Italy ranged from 0.7 per 100,000 children during 2010 to 2 per 100,000 children during 2022. The median time between the onset of thrombocytopenia and the diagnosis of ITs was 1 years (range 0 - 18 years). A family history of thrombocytopenia has been reported in 90 patients (65%). Among 139 children with ITs, in 73 (53%) children almost one defective gene has been identified. In 61 patients a pathogenic mutation has been identified. Among them, 2 patients also carry a variant of uncertain significance (VUS), and 4 others harbour 2 VUS variants. VUS variants were identified in further 8 patients (6%), 4 of which carry more than one variant VUS. Three patients (2%) had a likely pathogenic variant while in 1 patient (1%) a variant was identified that was initially given an uncertain significance but was later classified as benign. In addition, in 17 patients the genetic diagnosis is not available, but their family history and clinical/laboratory features strongly suggest the presence of a specific genetic cause. In 49 children (35%) no genetic defect were identified. In ninetyseven patients (70%), thrombocytopenia was not associated with other clinically apparent disorders. However, 42 children (30%) had one or more additional clinical alterations.

**Conclusion:**

Our study provides a descriptive collection of ITs in the pediatric Italian population.

## Introduction

Inherited thrombocytopenias (ITs) are a heterogeneous group of congenital bleeding disorders characterized by a reduced platelet count and variable clinical course. To date, a total of 45 different forms of ITs have been identified with different clinical expressions and variable prognosis. Main forms of ITs are exclusively characterized by a decreased platelet count with bleeding symptoms that vary in severity, ranging from severe clinical presentations, which may be revealed immediately after birth, to mild clinical presentations that could remain undiagnosed until fortuitous identification during routine laboratory examinations (1–3). Despite bleedings being considered the main clinical manifestation for patients with inherited thrombocytopenias, ITs are frequently associated with other congenital defects or an increased risk of developing further diseases such as hematological malignancies and kidney failure.

The prevalence of ITs in Europe is reported to be 2 per 1 million children (4). Although ITs are rare, latest advances in understanding these disorders suggested that their prevalence may be higher than previously thought (5, 6). Making a correct diagnosis of ITs may be difficult and often delayed because of the rarity of these conditions and their not specific clinical presentation. Moreover, ITs are often unrecognized and misdiagnosed with most common forms of thrombocytopenia. In addition, in more than of 50% of patients with ITs, the molecular cause remains unknown (7, 8).

To date, several studies describe the clinical picture and genetic characterization of ITs; they are usually focused on specific forms of ITs and report single-center case experiences (9, 10). However, larger further studies are needed to improve the clinical assessment and standardization of diagnosis of patients with ITs and to evaluate the efficacy of innovative therapeutic approaches (e.g., thrombopoietin receptor agonists) used successfully in acquired thrombocytopenias (11–13).

This multicenter retrospective–prospective study is the first one that provides a comprehensive overview of clinical, laboratory, and long-term outcomes of a large cohort of Italian children affected by ITs with the aim to improve knowledge and clinical management of these disorders.

## Material and methods

The “CHildren with Inherited Platelet disorders Surveillance” study (CHIPS) is a retrospective–prospective observational cohort study conducted between January 2003 and January 2022 in 17 centers affiliated to the Italian Association of Pediatric Hematology and Oncology (AIEOP).

The primary objective of this study was to collect clinical and laboratory data on Italian pediatric patients with ITs. Secondary objectives were to calculate the prevalence of ITs in the Italian pediatric population and to assess frequency and genotype–phenotype correlation of different types of mutations in our study cohort. Patients with inherited thrombocytopenia aged from 0 to 18 years in which the genetic defects have been identified and/or with a suggestive familiar history of thrombocytopenia were included in the study. Patients with thrombocytopenia due to other causes (e.g., neonatal, immune, oncological, or infective), aged over 18 years, and without an identifiable genetic defect and a suggestive familiar history of thrombocytopenia were excluded. Demographic data, family history, genetic variant, clinical characteristics, treatments, and laboratory findings were collected. Bleeding scores were assigned by clinicians using the Buchanan and Adix scoring system, as this is routinely used by our Italian working group (14). Thrombocytopenia was defined as a platelet count <150 × 10^9^/L. A platelet count between 150 and 450 × 10^9^/L was considered normal (15, 16). Genetic variants were classified by testing laboratories as pathogenic, likely pathogenic, variants of uncertain significance (VUS), likely benign, or benign/polymorphism, following the 2007 guidelines from the American College of Medical Genetic and Genomics (17).

Local ethics committee approval and written informed consent were obtained. The caregivers provided signed consent forms and data collection was performed according to Italian regulation for personal data protection.

### Mutational screening

The patients described in this manuscript were screened by different centers over a time span of nearly 20 years. In addition, the technologies used to screen for mutations have changed profoundly over the course of this study. Most patients enrolled between 2003 and 2017 were analyzed by Sanger sequencing with candidate gene approach based on the clinical characteristics of the patient and after application of the diagnostic algorithm described by Balduini et al. (18).

Since 2017, most patients were analyzed by the Medical Genetics Unit of IRCCS Burlo Garofolo Children's Hospital in Trieste by target sequence approach based on Ion Torrent Personal Genome Machine (Ion PGMTM) platform. Sequencing primers were designed on the coding and intronic flanking regions of 28 IT genes using the Ion Ampliseq Designer software (https://www.ampliseq.com) (Supplementary Table S2).

Following the manufacturer's recommendations (Life Technologies), two multiplex PCRs were carried out for each sample using the Ion AmpliSeq library kit 2.0. Emulsion-PCR and enrichment reactions were performed on the template using Ion One Touch 2 system. Sequencing reactions were performed using Ion PGMTM Sequencing 200 Kit v2. Sequencing data were analyzed using Ion Torrent Suite software (v.5.12). Data were aligned with hg19 human genomic sequence using the plug-in Variant Caller (TSVC v5.6 and v.5.12). Functional annotations of all the sequence variants were performed using the wANNOVAR software (http://wannovar.usc.edu/).

Only rare variants with a minor allele frequency <0.01 were considered for the analysis, and all variants reported were confirmed by Sanger sequencing using standard conditions in an ABI 3100 automated sequencer (Applied Biosystems, Foster City, CA, United States). Some patients underwent whole exome sequencing (WES) analyses.

### Interpretation of identified variants

The large amount of genetic data emerging from next-generation sequencing approaches has the consequence that the meaning attributed to identified variants can also change rapidly over time due to the frequency with which these variants are detected or by the fact that some variants are identified only in certain groups of individuals. Therefore, especially in cases where variants of uncertain significance have been identified, their interpretation must periodically be re-evaluated. This process is very important because it allows some variants to be reclassified over time.

All tools used to assess the pathogenicity of the variants identified in this work were again browsed and updated during the revision process of this manuscript in order to provide the most up-to-date interpretation. Variant pathogenicity was evaluated taking into account several criteria that do not always agree in classification. Specifically, it was assessed whether the variant had been functionally studied or had already been reported in the literature and/or classified as a cause of disease by the Human Gene Mutation Database (HGMD). In addition, interpretations from the InterVar and ClinVar software were also considered.

## Results

### Demographic and baseline data

**Figure 1:**
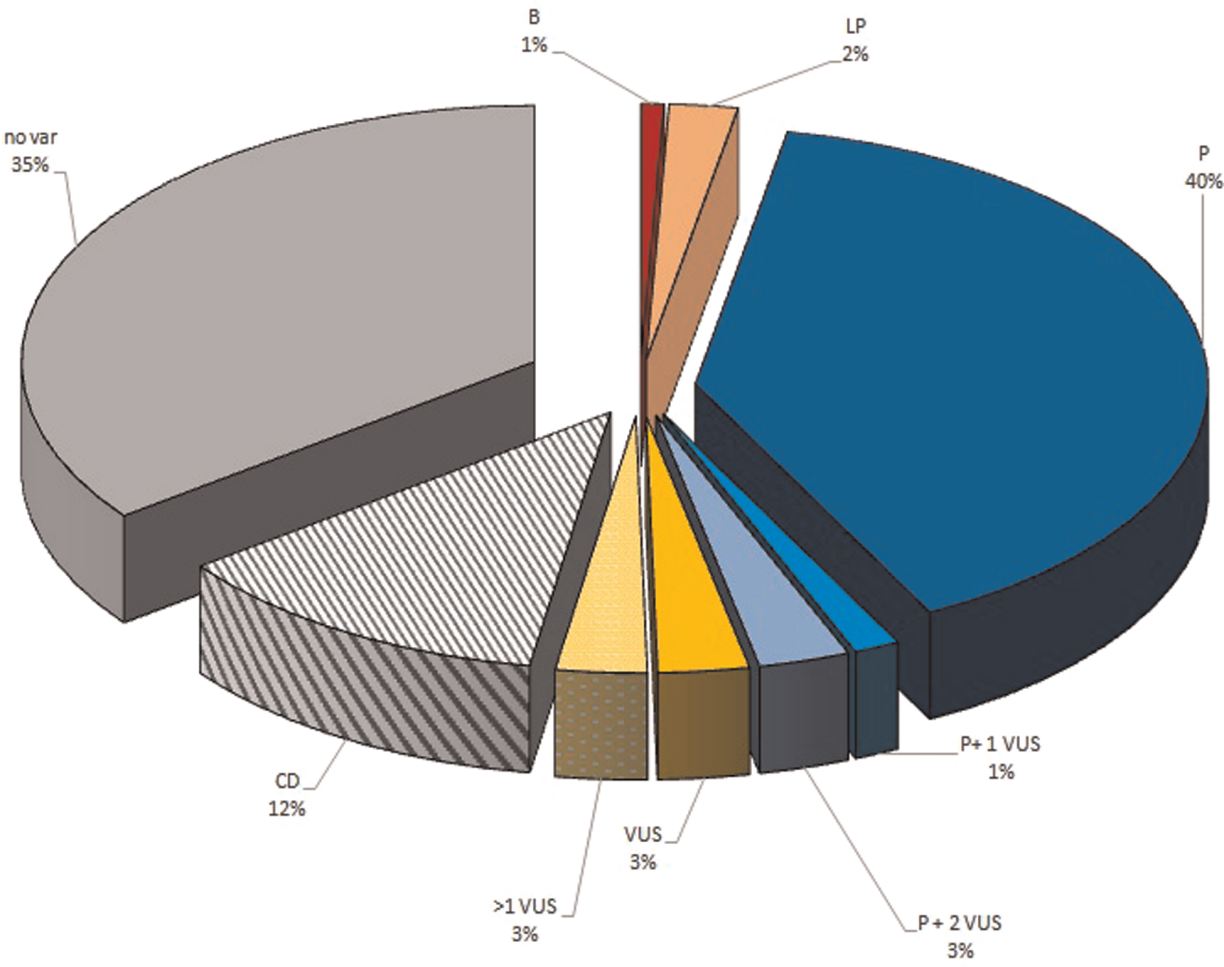
Graphical representation of IT genetic variants detected on the CHIPS cohort. B, benign variant; LP, likely pathogenetic variant; PV, pathogenetic variant; VUS, variant of uncertain significance; CD, clinical diagnosis based on clinical features or family history; no var, no candidate variants identified.

A total of 139 children with IT (82 males and 57 females) were enrolled. ITs prevalence in Italy ranged from 0.7 per 100,000 children during 2010 to 2 per 100,000 children during 2022. The median time between the onset of thrombocytopenia and the diagnosis of ITs was 1 year (range 0–18 years). Family history of thrombocytopenia has been reported in 90 patients (65%). The median time between the onset of thrombocytopenia and the diagnosis of ITs was 1 year in patients with a family history of thrombocytopenia (range 0–18 years) and 4 years in patients without a family history of thrombocytopenia (range 0–18 years). There are no statistically significant differences between the groups (*p* value: 0.6). Table 1 lists the demographic and baseline features of the included patients. Among 139 children with ITs, in 73 (53%) children, almost one defective gene has been identified. In 61 patients, a pathogenic mutation has been identified (Table 2). Among them, two patients also carry a VUS, and four others harbor two VUS variants. VUS variants were identified in further eight patients (6%), four of which carry more than one variant VUS. Three patients (2%) had a likely pathogenic variant, while in one patient (1%), a variant was identified that was initially given an uncertain significance but was later classified as benign (32). In addition, in 17 patients, the genetic diagnosis is not available, but their family history and clinical/laboratory features strongly suggest the presence of a specific genetic cause. In 49 children (35%), no genetic defects were identified. These results are summarized in Figure 1.

**Table 1:** Demographic and baseline features of the included patients.

Total		
* n*	139
Gender, *n* (%)		
M	82	(59%)
F	57	(41%)
Age at onset of thrombocytopenia, years		
Median (range)	4 (0–18)	
Baseline platelet count, ×10^9^/L		
Median (range)	82 (2–147)	
Familiar history of thrombocytopenia, *n* (%)		
Yes	90	(65%)
No	49	(35%)
Bleeding score (Buchanan and Adix bleeding score)		
Median (range)	0 (0–4)	
Therapies, *n* (%)		
Yes	46	(33%)
Intravenous immunoglobulin	15	(32%)
Platelet transfusion	12	(26%)
Tranexamic acid	12	(26%)
Corticosteroids	9	(19%)
Ferrous sulfate	6	(13%)
Red blood cell transfusions	5	(11%)
thrombopoietin receptor agonists	5	(11%)
Folic acid	2	(4%)
Estrogen and progestin oral contraceptive	2	(4%)
Recombinant factor VIIa	1	(2%)
Antimicrobial prophylaxis	1	(2%)
Tegretol	1	(2%)
Dilatrend	1	(2%)
Erythropoietin	1	(2%)
Mycophenolate mofetil	1	(2%)
No	93	(67%)

**Table 2:** Patients with pathogenetic variants.

Families	Patients	Gene	cDNA/*gDNA*	Protein/*RNA*	Status	References
1	I	*GP1BA*	c.515C > T	p.Ala172Val	het	Noris et al., 2012 (19)
	II				het	
2	I				het	
3	I				het	
4	I				het	
5	I				het	
6	I				het	
7	I				het	
8	I				het	
9	I				het	
10	I				het	
11	I				het	
12	I				het	
13	I				het	
14	I		c.104delA	p.Lys35Argfs*4	hom	Li et al., 1996 (20)
	II				hom	
15	I		c.217C>T	p.Leu73Phe	het	Miller et al., 1992 (21)
16	I	*MYH9*	c.221_223delAGA	p.Lys74del	het	Smith et al., 2019 (22)
17	I		c.279C>G	p.Asn93Lys	het	Seri et al., 2000 (23)
18	I		c.3493C>T	p.Arg1165Cys	het	Seri et al., 2000 (23)
	II				het	
19	I		c.4270G>C	p.Asp1424His	het	Seri et al., 2000 (23)
20	I		c.4270G>A	p.Asp1424Asn	het	Pecci et al., 2009 (24)
21	I		c.5521G>A	p.Glu1841Lys	het	Seri et al., 2000 (23)
22	I				het	
23	I		c.5797C>T	p.Arg1933*	het	Pecci et al., 2009 (24)
24	I	*ANKRD26*	c.-116C>T		het	Noris et al., 2013 (25)
25	I				het	
26	I		c.-128G>A		het	Pippucci et al., 2011 (26)
27	I				het	
28	I				het	
29	I				het	
30	I				het	
31	I	*ACTN1*	c.673G>A	p.Glu225Lys	het	Bottega et al., 2015 (27)
32	I		c.221°C>A	p.Thr737Asn	het	Faleschini et al., 2018 (28)
33	I	*CYCS*	c.124G>A	p.Gly42Ser	het	Morison et al., 2008 (29)
	II				het	
34	I		c.145T>C	p.Tyr49His	het	De Rocco et al., 2014 (30)
35	I				het	
36	I	*RUNX1*	c.334delC	p.Leu112Cysfs*10	het	
37	I		c.524dupT	p.Thr176Aspfs*37	het	
38	I		c.967+2_5del	*r.[967_968ins886 + 1_967 + 63; 967 + 2_5del]* (*p*.Ala297Hisfs*23)/*r.806_967del* (p.Asp269_Thr323delinsAla)	het	De Rocco et al., 2017 (31)
39	I		*21q22.12 (36331450_36945345)del (Jacobsen)*		het	
40	I	*ETV6*	c.1040A>C	p.Gln347Pro	het	Faleschini et al., 2022 (32)
41	I				het	
42	I	*PTPRJ*	c.97-2A>G		het	Marconi et al., 2019 (33)
			c.1875delG	p.Ser627Alafs8X	het	
	II		c.97-2A>G		het	
			c.1875delG	p.Ser627Alafs8X	het	
43	I	*WAS*	c.223G>A	p.Val75Met	het	Raimohan et al., 2009 (34)
44	I				het	
45	I		c.254T>C	p. Ile85Thr	het	Raimohan et al., 2009 (34)
46	I		c.397G>A	p.GLu133Lys	het	Raimohan et al., 2009 (34)
47	I		c.708delT		het	
48	i		c.778-6G>A		het	Albert et al., 2010 (35)
49	I		*11q del (Jacobsen)*		het	
50	I	*SRC*	c.1579G>A	p.Glu527Lys	het	Barozzi et al., 2020 (36)
51	I	*GNE*	c.1546_1547delinsAG	p.Val516Arg	hom	Bottega et al., 2021 (37)
52	I	*ITGA2B*	c.175°C>T	p.Arg584*	het	Tomiyama et al, 1995 (38)
53	I		c.3076C>T	p.Arg1026Trp	het	Kunishima et al., 2011 (39)
54	I	*MPL*	c.408delC	p.Ser137Valfs*29	het	
55	I		c.1904C>T	p.Pro635Leu	het	Tijssen et al., 2008 (40)
56	I		*1q21.1 microdel*		het	Tassano et al., 2015 (41)

### Clinical characteristics

Children’s median bleeding score was 0 (range 0–4). In ninetyseven patients (70%), thrombocytopenia was not associated with other clinically apparent disorders. However, 42 children (30%) had one or more additional clinical alterations. Immune disorders and/or recurrent infections (9%), cognitive impairment (8%), skeletal (4%) or otolaryngological abnormalities (5%), central nervous (4%) or cardiovascular system (4%) malformations, gastrointestinal (9%), dermatological (4%), ocular (3%), urogenital (5%), and endocrinological disorders (8%) were often associated with thrombocytopenia. Patients with pathogenic variants are described below.

### Mutation in *GP1BA*

Known mutation in the *GP1BA* gene was identified in 17 patients (12%) (7 males and 10 females). Among them, 14 patients from 13 families carry the c.515C>T (p.Ala172Val) mutation, also known as Bolzano mutation (19). In one of these patients (2-I), the Bolzano mutation was associated with the *ABCG8* variant classified as VUS. With regard to the remaining three patients, a homozygous small deletion (c.104delA), which has been previously associated with the Bernard–Soulier syndrome (BSS) (20), was identified in two individuals belonging to same family. In accordance with a diagnosis of BSS, these patients show increased mean platelet volume (MPV) as well as increased bleeding tendency (Supplementary Table S1). The pathogenic variant c.217C>T (p.Leu73Phe) was identified in the last patient as described in Table 2. The median age at the diagnosis was 3 years (0–13 years), while the median platelet count at the diagnosis was 93 × 10^9^/L (14–147 × 10^9^/L). The median bleeding score was 0 (range 0–3). Three patients (18%) (14-I, 14-II, 13-I) required one or more treatments (e.g., red cell concentrates transfusions, platelet transfusions, tranexamic acid). No patient (94%) had symptoms or laboratory abnormalities associated with thrombocytopenia except one patient (6%) affected by craniofacial dysmorphisms (Goldenhar syndrome) (2-I).

### *MYH9*-related thrombocytopenia

Thirteen patients (9%), 10 males and 3 females, were affected by *MYH9*-related thrombocytopenia. Pathogenic mutations in the *MYH9* gene were identified only in nine patients from eight different families (Table 2). In one patient (17-I), mutation of *MYH9* was associated with two *VWF* variants classified as VUS. Although in the remaining four patients the genetic diagnosis is not available, their family history and clinical/laboratory features allowed us to include them in the *MYH9*-related thrombocytopenia patients. The median age at the diagnosis was 5 years (0–13 years) while the median platelet count at the diagnosis was 50 × 10^9^/L (9–90 × 10^9^/L). The median bleeding score was 1 (range 0–3). Three patients (23%) required one or more treatments. Among 13 patients with *MYH9*-related thrombocytopenia, 10 patients (77%) were asymptomatic, 1 patient (8%) suffered from sensorineural hearing loss, 1 patient (8%) had chronic renal failure and neurobehavioral disorders (19-I), and 1 patient (18-I) (8%) had facial dysmorphisms and Hirschsprung’s disease.

### *ANKRD26*-related thrombocytopenia

*ANKRD26*-related thrombocytopenia was detected in nine patients (6%) (five males and four females). Although a known mutation was identified in seven patients (Table 2), the family history (presence of the genetic mutation in the family, thrombocytopenia inherited with an autosomal dominant pattern, and history of myelodysplastic syndrome and/or myeloid neoplasms) and clinical/laboratory features of the other two patients strongly suggest the presence of a genetic cause. In one patient (30-I) without significant clinical and laboratory characteristic, *ANKRD26* mutation was associated with *GP1BA* and *NBEAL2* variants classified as VUS. Among patients with *ANKRD26*-related thrombocytopenia, the median age at the diagnosis was 7 years (3–15 years) while the median platelet count at the diagnosis was 58 × 10^9^/L (41–115 × 10^9^/L). The median bleeding score was 0 (range 0–3). Four patients (44%) (29-I, 25-I, 24-I) required one or more treatments. Among nine patients with *ANKRD26*-related thrombocytopenia, one patient (25-I) (11%) developed myelodysplasia at the age of 10. Exitus occurred after hematopoietic stem cell transplantation (HSCT) owing to complications of severe idiopathic pneumonia syndrome.

### *WAS*-related thrombocytopenia

*WAS*-related thrombocytopenia was detected in 12 patients (7%) (eleven males and one female). Among them only six patients obtained a molecular diagnosis (Table 2), while in six patients for whom the genetic data are not available, the diagnosis was based on their family history and clinical/laboratory features. The median age at the diagnosis was 4 years (0–8 years) while the median platelet count at the diagnosis was 29 × 10^9^/L (13–63 × 10^9^/L). The median bleeding score was 0 (range 0–3). Ten patients (83%) required one or more treatments. Among 12 patients with WAS-related thrombocytopenia, 8 patients (66%) had no symptoms or laboratory abnormalities associated with thrombocytopenia. One patient (48-I) (8%) was affected by splenomegaly, immunodeficiency, otolaryngological alterations, and central nervous abnormalities; another patient (8%) developed dermatological alterations, ischemic stroke at the age of 4 years, and hepatic lymphoblastic lymphoma at the age of 9 years. He had undergone HSCT with complete remission of the disease. In addition, one patient (47-I) (9%) at birth had experienced cephalohematoma and cholestasis. He had undergone HSCT at the age of 6 months and died of complications. Two patients (44-I) (16%) had undergone HSCT at the age of 2 years with complete remission of the disease.

### *ACTN1*-related thrombocytopenia

*ACTN1*-related thrombocytopenia was diagnosed in three male patients (2%). As reported in Table 2, two of them carry a known pathogenic mutation, while in the remaining patient, the diagnosis was possible due to the clinical/laboratory features and because the diagnosis of *ACTN1*-RT was reported in the patient's father. The median age at the diagnosis was 3 years (2–11 years) while the median platelet count at the diagnosis was 97 × 10^9^/L (87–104 × 10^9^/L). In all patients, bleeding symptoms were mild (median bleeding score 1, range 0–1). Among three patients with *ACTN1*-related thrombocytopenia, one patient (33%) was asymptomatic. One patient (33%) had a congenital abnormality of the urinary tract (vesicoureteral reflux), while one patient (32-I) (33%) had splenomegaly and gallbladder anomalies.

### *CYCS*-related thrombocytopenia

A known pathogenic mutation in the *CYCS* gene was identified in four male patients (3%), two of them belonging to the same family (Table 2). The median age at the diagnosis was 10 years (3–18 years) while the median platelet count at the diagnosis was 124 × 10^9^/L (103–144 × 10^9^/L). All children presented mild bleeding symptoms without additional symptoms or laboratory abnormalities associated with thrombocytopenia.

### *RUNX1*-related thrombocytopenia

*RUNX1* mutations were identified in four patients (3%) (two males and two females). Of note, mutation c.967+2_5del, generates three different RNA transcripts and two different protein products as reported in Table 2. The pathogenetic effect of this mutation is reported in detail by De Rocco and colleagues as well as the association of c.967+2_5del with impaired platelet aggregation (30). Defects in platelet aggregation were also observed in patient 39-I in whom a deletion of about 600 kb, resulting in the lack of exons 1 and 2 of the *RUNX1* gene, was detected. The median age at the diagnosis was 6 years (3–9 years) while the median platelet count at the diagnosis was 84 × 10^9^/L (44–141 × 10^9^/L). None of the patients reported bleeding events (median bleeding score: 0, range 0–0). Among four patients with *RUNX1*-related thrombocytopenia, two patients (50%) had not abnormalities associated. Thrombocytopenia, cognitive impairment, and thyroid dysfunction were reported in one patient (37-I) (25%). In addition, at the age of 17, one patient (39-I) (25%) developed a myelodysplastic syndrome, a successively acute myeloid leukemia. She had undergone HSCT with complete remission of the disease.

### *ETV6*-related thrombocytopenia

*ETV6*-related thrombocytopenia was detected in two patients (1%) (one male and one female). Of note, the two patients, belonging to two unrelated families, carry the same mutation, recently classified as pathogenic (32). The median age at the diagnosis was 1 year (0–12 years) while the median platelet count at the diagnosis was 61 × 10^9^/L (17–97 × 10^9^/L). The median bleeding score was 0 (range 0–0). Both patients required one or more treatments. One patient (41-I) (50%) had cognitive impairment, epilepsy, and dyslexia.

### *PTPRJ*-related thrombocytopenia

Whole exome sequencing performed in two siblings (1%) (9-year-old male and 17-year-old female) allowed us to identify two compound heterozygous mutations in *PTPRJ* gene, responsible for a novel form of thrombocytopenia (33). The median platelet count at the diagnosis was 86 × 10^9^/L (77–96 × 10^9^/L). The girl had a history of spontaneous bleedings consisting in menorrhagia, easy bruising, petechiae, and epistaxis, resulting in mild iron deficiency anemia. The boy also presented spontaneous bleeding though of a milder degree. Except for bleeding tendency, medical history of both probands was unremarkable, and physical examination did not reveal any relevant abnormalities.

### Other pathogenic mutations

Genetic analysis allowed us to identify six further pathogenetic mutations in other five thrombocytopenia related genes, which are shown in Table 2 and are described as follows.

11q23 deletion (Jacobsen syndrome) was found in a 3-year-old girl with multiple cardiovascular and skeletal abnormalities and vascular malformations (aberrant subclavian artery). In this patient, thrombocytopenia was mild (100 × 10^9^/L at the diagnosis) without bleeding symptoms (Bleeding score: 0).

The pathogenetic microdeletion 1q21.1, associated with the thrombocytopenia-absent radius (TAR) syndrome, was detected in heterozygous state in a new-born with multiple skeletal, otolaryngological, and ocular anomalies and severe bleeding tendency. The TAR syndrome is a recessive form of thrombocytopenia caused by the combination of the 1q21.1 microdeletion in association with specific pathogenic single nucleotide polymorphisms (SNPs) in the other allele of the *RBM8A* gene; therefore, the alteration identified is not sufficient to obtain a molecular diagnosis. However, the clinical phenotype of this patient strongly suggests the presence of the second pathogenic mutation in the *RBM8A* gene that has not yet been identified.

Thrombocytopenia caused by a mutation in the *SRC* tyrosine kinase gene was found in an infant who suffered intracranial hemorrhage during birth. At 3 years of age, he developed myelodysplasia and had undergone HSCT complicated by acute graft versus host disease.

In patient 51-I reported in Table 2, the homozygous c.1546_1547delinsAG mutation in the *GNE* gene was associated with severe thrombocytopenia (median platelet count: 5 × 10^9^/L) and moderate hemorrhagic phenotype (bleeding score: 2).

The pathogenetic c.3076C>T (p.Arg1026Trp) mutation in *the ITGA2B* gene was detected in one patient with a mild thrombocytopenia (median platelet count: 90 × 10^9^/L) and hemorrhagic phenotype (bleeding score: 1). Of note, this patient also carried two *VWF* variants classified as VUS.

The pathogenetic c.175°C>T (p.Arg584*) mutation in the *ITGA2B* gene was detected in one patient (52-I) with a moderate thrombocytopenia (median platelet count: 18 × 10^9^/L) and with significant bleeding episodes (bleeding score: 2).

Finally, *MPL* mutations (55-I) were identified in one patient who developed trilinear cytopenia and undergone HSCT at the age of 1 year with complete remission of the disease and in another one with thyroiditis and mild thrombocytopenia (median platelet count: 63 × 10^9^/L). In the latter patient, *MPL* mutation (54-I) was detected together with a *NBEAL2* variant classified as VUS.

### Follow-up and outcome

In all patients, at least one clinical and laboratorial control was performed annually (range, 1–3). Follow-up valuation included (1) clinical assessment (100%); (2) complete blood count and reticulocyte count (100%); (3) peripheral blood smear (25%); (4) biochemical measurements of renal and hepatic function, electrolytes, and serum protein (20%); (5) urinalysis (5%); and (6) immunological assessment (3%), audiological evaluation (2%), and bone marrow examination (4%). The median time of follow-up was 2 years and 4 months (range, 0–16). Overall, 50 patients (35%) developed mucosal bleeding (22%) and cutaneous bleeding (13%), 4 patients (3%) developed myelodysplasia, and 7 patients (5%) had undergone HSCT. Among these patients, four patients had a complete remission of the disease, one patient had an acute graft vs. host disease, and two children died of complications.

## Discussion

ITs have been considered for a long time extremely rare diseases characterized by severe and life-threatening hemorrhagic symptoms. In the last few decades, considerable progress has been made in the understanding pathophysiology and molecular basis of ITs. To date, a total of 45 different forms of ITs have been identified with different clinical expression and variable prognosis (15, 42, 43). However, ITs are poorly known by clinicians and often misdiagnosed with most common forms of thrombocytopenia. The complexity of laboratory investigations available in few centers and the limited clinical experience in the knowledge of these forms are the most common causes of delayed diagnosis. Making an incorrect diagnosis exposes many patients to a suboptimal clinical management and useless therapies (44). Moreover, several forms of ITs predispose to additional complications, such as hematological malignancies or renal failure, which can be avoided with appropriate and timely treatment (45–49). Our retrospective–prospective multicenter study was the first study conducted in a large cohort of children of Italian population with the aim of improving knowledge about ITs. We found that the prevalence of ITs in the Italian pediatric population is higher with respect to previous data (4) and has increased significantly during the last few years. The progressive increase in the annual prevalence of ITs could be related to the improvement in knowledge of these disorders and the better ability to precociously identify them. In accordance with the possibility that diagnosis may be frequently complex and delayed, we found that the median time between the initial finding of thrombocytopenia and the diagnosis of inherited form was widely variable. The presence of a family history of thrombocytopenia, atypical features on the blood film, or associated diseases could lead to a prompt diagnosis (49). Although the median time to diagnosis appears lower in the group of patients with a family history of ITs compared to the group of patients without a family history, this difference is not statistically significant. This could be explained by the fact that patients with a familiar history of thrombocytopenia and a slightly lower-than-normal platelet count could consider medical intervention unnecessary and superfluous. Platelet count, genetic diagnosis, and clinical presentation varied considerably among the patients studied, which is consistent with the variability observed in the spectrum of ITs (42). We found that *GP1BA-*, *MYH9-*, *ACTN1-*, and *ANKRD26-*related thrombocytopenias are the most frequent diseases diagnosed with a highly variable clinical course and long-term prognosis. Their clinical and laboratory features are well recognized. According to several studies (50–52), we detected that in most patients, the mutation of *GP1BA* is exclusively associated with a macrothrombocytopenia. However, we reported in one patient multiple cardiovascular and skeletal dysmorphisms. Recently, Souto Filho et al. reported a case of Bolzano mutation associated with clinical features of 22q11.2 deletion syndrome with phenotypic spectrum of DiGeorge syndrome. This association could be explained by the fact that the constitutional hemizygosity of 22q11.2 may unmask an autosomal recessive disorder caused by alterations of the nondeleted *GP1BA* allele (53). Furthermore, we described in patients with MYH9-related thrombocytopenia neurobehavioral disorders, Hirschsprung’s disease, and facial dysmorphisms. In addition, congenital abnormality of the urinary tract, cardiac valve diseases, splenomegaly, and gallbladder anomalies were reported in two patients affected by *ACTN1*-related thrombocytopenia. The clinical significance of these abnormalities and their correlation with underlying platelet defect is still unknown, and future follow-up will be required.

As several forms of ITs are characterized by enlarged platelets, it is commonly recognized that the evaluation of platelet size is an important tool to suspect these diseases. In our study, we found that the MPV reported by the different centers is widely variable and not entirely correlated with the expected platelet size. However, the measurements of platelet size in ITs present substantial difficulties. As reported by Noris et al., the sensitivity and specificity of MPV in establishing platelet size is greatly variable depending on the instrument used. Furthermore, some of ITs (i.e., MYH9 and Bernard–Soulier syndrome) may present platelets that, due to their increased size, are unrecognized by the electronic counter, which therefore underestimates the MPV (54). In the last few years, new genes and *de novo* mutations responsible for inherited thrombocytopenia are continuously detected, and the classification of hereditary thrombocytopenias is updated constantly (55, 56). Therefore, pathogenicity could be due to different predisposing genetic variants in a polygenic setting. The use of next-generation sequencing (NGS) like whole genome and WES allowed the identification of causal genetic variants in both well-known and new genes involved in ITs, for example, *SLFN14*, *FYB*, *STIM1*, *GFI1B*, *ETV6*, and *PTPRJ*, but molecular mechanisms of some variants still remain unclear (31, 33, 57, 58). Although our knowledge regarding the causes of IT continues to grow, a genetic diagnosis is only reported in approximately 50% of patients and frequently variants of uncertain significance are detected (59). Recently, Johnson et al. analyzed 31 patients with ITs using whole exome sequencing. A variant of uncertain significance was identified in 51% of patients, while in 23% of patients, no variants have been detected (60). According to these studies, we observed that in less than half of IT patients, a pathogenic genetic defect have been identified. In addition, we reported that in 13% of patients, variants of uncertain significance have been detected, while in 33% of patients, no genetic defects were identified. Among variants of uncertain significance, we detected that *NBEAL2* gene mutation is the most frequent. We found highly variable clinical pictures in these patients with a wide variety of diseases associated with thrombocytopenia such as splenomegaly, central nervous system involvement, behavior impairment, and progressive bone marrow alterations. The molecular mechanism that explains the variable clinical presentation still remains undefined. Bottega et al. compared the clinical features of 11 patients with gray platelets syndrome. In these patients, a worse clinical course was seen in individuals with biallelic *NBEAL2* mutation. Moreover, in more than half of the patients, no gene alterations were identified suggesting that other defects in alternative genetic pathway are responsible for their platelet phenotype (61, 62). According to these studies, we found that in numerous patients, a genetic mutation was not detected or partially explained the pathogenic mechanism, although the clinical picture and anamnestic features are indicative of ITs.

Although further investigations are required to identify the genetic variations responsible for thrombocytopenia, the benefit of distinguishing ITs from acquired forms could become a critical step in improving patient clinical management and follow-up (63–66). Close monitoring including periodic clinical and laboratory examination could provide clinicians with greater knowledge of forms of thrombocytopenia without genetic identification.

In conclusion, our study provides a descriptive collection of the diagnosis of ITs in the pediatric Italian population. Despite the rarity of these hereditary disorders, collecting clinical and laboratory data and following patients over time could increase the knowledge of ITs and allow clinicians to diagnose them promptly and avoid further complications.

## Data Availability

The original contributions presented in the study are included in the article/supplementary material, further inquiries can be directed to the corresponding author/s.
